# Association Mapping Based on a Common-Garden Migration Experiment Reveals Candidate Genes for Migration Tendency in Brown Trout

**DOI:** 10.1534/g3.119.400369

**Published:** 2019-07-09

**Authors:** Alexandre Lemopoulos, Silva Uusi-Heikkilä, Pekka Hyvärinen, Nico Alioravainen, Jenni M. Prokkola, Chris K. Elvidge, Anti Vasemägi, Anssi Vainikka

**Affiliations:** * Department of Environmental and Biological Sciences, University of Eastern Finland, P.O. Box 111, FI-80101 Joensuu, Finland; † Department of Biology, University of Turku, FI- 20014, Turku, Finland; ‡ Department of Biological and Environmental Science, University of Jyväskylä, P.O. Box 35, FI-40014, Jyväskylä, Finland; § Natural Resources Institute Finland, Manamansalontie 90, FI-88300, Paltamo, Finland; ** Institute of Integrative Biology, University of Liverpool, Bioscience building, Crown street, L69 7BZ Liverpool, UK; †† Department of Aquaculture, Institute of Veterinary Medicine and Animal Sciences, Estonian University of Life Sciences, 51014 Tartu, Estonia, and; ‡‡ Swedish University of Agricultural Sciences, Department of Aquatic Resources, Institute of Freshwater Research, 17893 Drottningholm, Sweden

**Keywords:** Life-history strategies, RADseq, GWAS, salmonids

## Abstract

A better understanding of the environmental and genetic contribution to migratory behavior and the evolution of traits linked to migration is crucial for fish conservation and fisheries management. Up to date, a few genes with unequivocal influence on the adoption of alternative migration strategies have been identified in salmonids. Here, we used a common garden set-up to measure individual migration distances of generally highly polymorphic brown trout *Salmo trutta* from two populations. Fish from the assumedly resident population showed clearly shorter migration distances than the fish from the assumed migratory population at the ages of 2 and 3 years. By using two alternative analytical pipelines with 22186 and 18264 SNPs obtained through RAD-sequencing, we searched for associations between individual migration distance, and both called genotypes and genotype probabilities. None of the SNPs showed statistically significant individual effects on migration after correction for multiple testing. By choosing a less stringent threshold, defined as an overlap of the top 0.1% SNPs identified by the analytical pipelines, *GAPIT* and *Angsd*, we identified eight candidate genes that are potentially linked to individual migration distance. While our results demonstrate large individual and population level differences in migration distances, the detected genetic associations were weak suggesting that migration traits likely have multigenic control.

Genome-wide association studies (GWAS) aim to reveal links between genotypes and phenotypes. Originally developed for case-control comparisons in medical sciences (Ku *et al.* 2010), association mapping has been subsequently adopted for use on other organisms and for addressing agricultural, evolutionary and ecological questions. Recent studies have described genetic determinants for economically and ecologically important traits. For example, *vgll3* locus affects maturation age in Atlantic salmon *Salmo salar* in sex-dependent fashion (Ayllon *et al.* 2015; Barson *et al.* 2015) and *greb1l* affects migration timing in Pacific salmonids *Onchorynchus spp*. (Prince *et al.* 2017). These studies have revealed that traits that were traditionally thought to be influenced by tens or hundreds of genes (Waples *et al.* 2004; Johnston *et al.* 2014; Gutierrez *et al.* 2015) can actually be influenced by loci with major effect. Identification of these genotype-phenotype associations have helped to further understand the evolution of these traits in response to both natural and human-induced selection pressures (Czorlich *et al.* 2018).

Salmonids display a variety of anadromous and potamodromous migratory strategies with populations ranging from fully resident to fully migratory (Klemetsen *et al.* 2003; Chapman *et al.* 2012; Dodson *et al.* 2013). Prior to migration, some juvenile salmonids usually undergo a series of physiological and morphological changes, known as smoltification, that prepare the fish for seawater migration and entry to novel environments (Folmar and Dickhoff 1980; McCormick 2009; McCormick *et al.* 2013). During recent years, the genetic components underlying the dichotomy between resident and migratory forms have been increasingly studied, particularly in the genus *Onchorhynchus* (*e.g.*, Hale *et al.* 2013; Nichols *et al.* 2016; Veale and Russello 2017). A single genomic region in chromosome 5 has been linked to the migration differences between resident “rainbow trout” and migratory “steelhead” populations of *O. mykiss* (Hecht *et al.* 2012; Pearse *et al.* 2014; Leitwein *et al.* 2017a), yet not in all (Hale *et al.* 2013). In addition, an extensive list of candidate genes for migration propensity has been identified for *O. mykiss* (Hecht *et al.* 2013; Hess *et al.* 2016) as well as for other species within the genus (Nichols *et al.* 2016; Veale and Russello 2017). To obtain a representative view of genetic variants influencing propensity to migrate, genome wide markers with as good coverage as possible should optimally be used. However, any significant indications for genetic control would be important in answering the question whether migration propensity can evolve in response to natural and human-induced selection pressures.

Brown trout *Salmo trutta* is an ecologically and economically important salmonid native to Europe, Asia and Northern Africa, and occurs currently as migratory, resident and partially migratory populations in all continents except for Antarctica (MacCrimmon and Marshall 1968). Migratory (anadromous and potamodromous) populations are frequently threatened by anthropogenic factors such as dam building and overfishing, while many resident populations are often isolated and occur mainly in small headwaters with lesser human impact. It is crucial to gain knowledge about the underlying causes of migration in brown trout to understand the historical and contemporary interactions between resident and migratory forms, and resolve the evolvability of migration tendency in both hatchery breeding and fisheries that may impose selection on migration traits. Traditionally, brown trout have been considered an extreme example of phenotypic plasticity when it comes to migration, with food availability and conspecific density reportedly driving migratory behavior in empirical field-studies (Olsson *et al.* 2006; Wysujack *et al.* 2009; Kendall *et al.* 2015). However, by comparing migratory and resident brown trout populations using genome-wide genetic markers, we have recently identified a subset of outlier genes that potentially influence migration propensity (Lemopoulos *et al.* 2018). However, the extent of genetic control over this trait, as well as whether a common basis for migration exists among different populations of brown trout or among salmonids, has yet to be determined (Ferguson *et al.* 2019).

Studying individual-level migration patterns in wild populations is challenging for a number of reasons, including high cost, intensive workload and potential negative effects of telemetry tags on focal animals (Thorstad *et al.* 2013). Moreover, natural populations are subject to different environmental pressures and any genetic signatures of selection can reflect processes that are independent of, or completely confounded with, the adaptations directly linked to migration. Experimental common-garden designs can overcome these challenges by providing uniform environmental conditions for all genotypes (Liedvogel *et al.* 2011; de Villemereuil *et al.* 2015). Migration tendency consists of overall probability to migrate (migration propensity) and eventual migration distance. Because migration tendency can be a difficult trait to measure *per se*, experimental studies have often focused on smoltification-related traits such as growth, condition, body coloration, morphology and osmoregulatory enzyme activities (Nichols *et al.* 2008; Hecht *et al.* 2012; Baerwald *et al.* 2016). Despite the potential advantage of using a common-garden design to create uniform environmental conditions and the power of genome-wide markers to reveal genetic determinants of migratory behavior, only few studies to date have successfully combined these two approaches (*e.g.*, Nichols *et al.* 2008; Narum *et al.* 2011; Hecht *et al.* 2014).

Here, we performed a multi-year common garden experiment to investigate the genetic basis of migration tendency, measured as migration distance in brown trout. We artificially propagated brown trout from one predominantly migratory and one predominantly resident population (Lemopoulos *et al.* 2018) originating from a single water system using a replicated full factorial 3 males × 3 females matrix design, and continuously followed the movements of F_1_ individuals over two smolt migration seasons at the ages of two and three years. Restriction site associated DNA sequencing (RADseq) was used to genotype 116 individuals from the tails of the estimated distribution of migration distances (*i.e.*, individuals showing the longest and shortest migration distances). We used two complementary association analyses to identify candidate genes for migration tendency in data corrected for population differences. Since brown trout migratory behavior has been recently found to associate with several outlier markers (Lemopoulos *et al.* 2018), we anticipated that the outliers and/or genomic regions identified by the earlier genome scan would associate with the experimentally determined individual migration distance. Thus, we aimed to a) identify novel candidate genes that would explain individual migration distance, and b) validate the functional links between previously detected and overlapping outlier SNPs and migration tendency.

## Material and Methods

### Common garden experiment

We used two strains of brown trout originating from a single river system discharging to Lake Oulujärvi, Northern Central Finland, for this study: 1) hatchery-bred migratory fish originating from rivers Varisjoki and Kongasjoki (referred as OUV), and 2) wild resident fish collected via electrofishing from a small headwater tributary (Vaarainjoki, VAA; see Lemopoulos *et al.* 2018). The wild fish were transported and held in seminatural ponds at the Kainuu Fisheries Research Station (Natural Resources Institute Finland (LUKE): www.kfrs.fi; see Lemopoulos *et al.* 2019) until artificial fertilization on 16^th^ October 2013. The wild (females: *n* = 9, length 357 mm ± 30.8 mm (mean ± SD), mass 520.3 g ± 126.0 g; males: *n* = 9, length 400.4 mm ± 82.3 mm, mass 795.8 g ± 412.0 g) and hatchery (*n* = 9 males and 9 females, year class 2008, individual sizes missing, mean weight 1794 g) fish were crossed in fully factorial 3 × 3 matrices in three replicates (within and between population crosses). The eggs were pooled within strains (equal proportions between families) and incubated in four replicates per strain. After hatching, the progeny was raised in the hatchery in 3.2 m^2^ fiberglass tanks (four replicate tanks per strain, 1595-2109 fish per tank on 20^th^ February 2014) according to standard methods and fed *ad libitum* with size-adjusted commercial dry feeds (Raisioagro, www.raisioagro.com) until tagging (Hyvärinen and Rodewald 2013). On 18^th^ September 2015, twenty individuals were randomly dip-netted from each rearing tank (*n* = 80 per strain including the hybrid strain, *n* = 240 total) and tagged with a 12 mm half duplex passive integrated transponder (HDX-PIT) tags under benzocaine anesthesia (40 mg L^−1^). The fish (mean length ± SD 159.9 mm ± 18.1 mm, mean body mass ± SD 47.5 g ± 16.7 g) were randomized evenly into eight circular migration channels (*n* = 10 per group, *n* = 30 per channel) (Fig. S1).

The experimental migration channels (width 1.5 m, mean length 26.15 m) were built in 75 m^2^ circular outdoor concrete ponds with dark green plastic tarpaulin tent covers allowing natural but shaded light conditions inside the tanks (Fig. S1). The water input (∼55 l s^-1^) was adjacent to the water outlet to create unidirectional flow (average depth 0.3 m, water flow ∼0.11 m s^-1^). The channels were equipped with four stationary PIT-tag antennae (reading rate 9 s^-1^) at equal intervals. An antenna was constructed of five coil inductor loops made of PVC-coated multithread copper wire (ø = 4 mm) (1350 mm × 300 mm, length × width), with each antenna connected to one of four computers configured to run and save (TIRIS datalogger program, Citius solutions Oy 2009) ASCII data (for further details see Janhunen *et al.* (2011)).

### Smolt experiment with 2-year old fish

**Figure 1:**
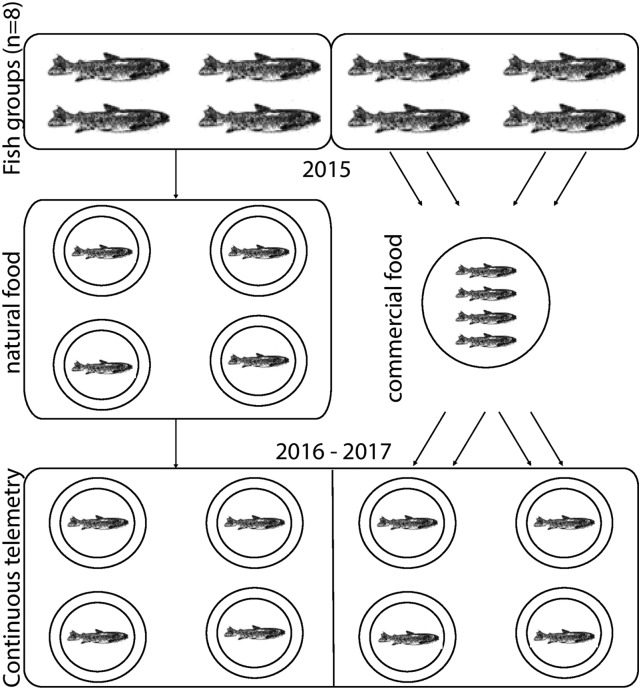
Diagram of the rearing of 8 fish groups used for a 2-year common garden experiment.

Because smolt migration is expected to be influenced by food availability (Vainikka *et al.* 2012), we used two feeding regimes: half of the fish groups (*n* = 4) were transferred to the experimental channels supplied only with natural food on 5^th^ November 2015 (Figure 1), and the rest of the groups (*n* = 4) were transferred into a 50 m^2^ concrete rearing pond in order to be fed with commercial fish feeds *ad libitum* until April 2016. The feed-augmented fish (and one VAA fish tagged on 25^th^ February 2016 replacing one accidental mortality) were recovered on 14^th^ April 2016 and transferred to the four experimental channels the next day. Continuous PIT-telemetry in all eight streams (see also Vainikka *et al.* 2012 for more details) was started on 16^th^ April 2016 at midnight and continued until the end of the experiment at midnight on 26^th^ June 2016. The fish were measured for body length and body mass and sampled for a piece of caudal fin tissue for DNA extraction under anesthesia on 28^th^ June 2016. After the measurements, four random samples of thirty fish within the natural food treatment (amended with three fish from the spring stocking group to replace mortalities) were returned back to the smolt migration ponds where they were maintained with natural food until the end of the experiment in following summer 2017. The rest of the fish were transferred to a 50 m^2^ concrete pond to be maintained with commercial dry food until the spring release in 2017 (see below).

### Smolt experiment with 3-year-old fish

Brown trout augmented with commercial feed tested in 2016 (*n* = 115 + five reserve fish with no previous test) were held in a 50 m^2^ concrete rearing ponds with additional fish from the same cohort (*n* = 444 in total) over winter. The fish were recovered and measured for length (to 1 mm, mean ± SD 275.2 ± 32.2 mm) and body mass (to 0.1 g, mean ± SD 238.7 ± 91.0 g) under anesthesia on 14^th^ March 2017. These fish were then transferred into four migration channel ponds after one night’s recovery in four 3.2 m^2^ indoor fiberglass tanks on 15.3.2017 at 10:00 (ten fish per strain, *n* = 30 per pond). The migration experiment was ended at two occasions: one random pond per feeding regime was emptied on 7^th^ June 2017 to collect lethal physiological samples from killed fish and the rest of the ponds were emptied on 21^th^ June 2017. All recovered fish were measured for length and body mass (within the subset of sequenced fish, OUV: 267.2 mm ± 62.4 mm (average ± SD), 211.7 g ± 139.7 g; VAA: 231.6 mm ± 39.8 mm, 123.2 g ± 60.8 g) under anesthesia, and fish in good condition were transferred back to the rearing ponds under standard rearing and feeding protocols. The fish sampled on 7^th^ June 2017 were analyzed for their migration behavior from 16^th^ March 2017 at midnight to 6^th^ June 2017 at midnight. All the other fish were analyzed for their migration behavior from 16^th^ March 2017 at midnight to 20^th^ June 2017 at midnight.

### Quantification of individual migration tendency

PIT detection data were transformed to individual movements per hour by counting the PIT-specific detections at each antenna using application-specific software (by Niko Vuokko, http://pitdata.net/). Based on the order of detections, the movement distances were calculated for each individual as quarter rounds up- and downstream. The hourly data were further analyzed by calculating the total individual distance moved downstream using custom codes in AV Bio-Statistics 5.2 (written by A.V.). The recording computers had to be restarted on a weekly basis, and when calculating migratory distances, the fish were assumed to have maintained their previous position during these maintenance breaks (< 30 min).

To maximize phenotypic variance between the limited number of samples that could be sequenced, we initially chose the four most and least migratory individuals (based on total downstream distance on equal time periods) in each channel (*n* = 128) and prioritized the presence of data from both years. This selection procedure resulted a subset of 116 individuals (out of 160 in total, n = 58 from both strains) that is not representative of all individuals in the experiment but provided extreme phenotypic values for the GWAS. Because individual migration distances could have been influenced by the test channel (which was confounded with diet treatment), rearing tank, year, sex (see below for the determination method) and feeding treatment, we removed their effects using a linear mixed effects (LME) model including sex, year (also as repeated), test pond and year × pond –interaction as fixed factors and rearing tanks / test pond during the previous year as a random factor. Prior to the analysis, migration distances were ln-transformed to meet normality. The model was fitted in SPSS 23.0.0.2 (IBM Corp.) using restricted maximum likelihood with random terms based on variance components and temporal covariance matrix based on diagonal structure with heterogenous variance. The residuals from this model were used to represent individual tendency to migrate (Fig. S2). The values were comparable between the channels, sexes and years, but included the effect of strain and fish size as these variables could mechanistically explain the originally genetic effect on migration. For the GWAS, the residual values for the two years were averaged; when migration distance was available for one year only (*n* = 12 fish, including five migratory strain fish that died during 2016 and seven residents that died during 2017), that value was used. This was justified as the inter-year averages were zero and migration residuals showed high individual repeatability between years (ICC = 0.608, 95% C.I. 0.473 – 0.714, ICC package in R environment (Wolak *et al.* 2012)). To test the effect of strain on the phenotypic migration distance, we also fitted a model with strain as an additional fixed factor.

### Library preparation and genotyping

DNA samples were extracted from the 116 individuals using Macherey-Nagel NucleoSpin Tissue kit and quantified by fluorescence using Qubit 2.0. Sex of individuals was obtained through amplification and gel visualization of the sexually dimorphic *sdY* locus (Quéméré *et al.* 2014). *Sbf1* restriction enzyme was used in the library preparation, and the single-end sequencing was performed on two lanes on an Illumina HiSeq 3500 platform. Final DNA concentration measures, library preparation and sequencing were obtained from a commercial provider, Plateforme MGX - Montpellier GenomiX (Montpellier, France) that delivered the sequences in FastQ format for bioinformatics analyses. *Process_radtags* function in *Stacks* v1.40 software (Catchen *et al.* 2013) was used to demultiplex, quality check and clean the data. Reads of 120 base pairs (average of 1643201 reads per individual, average 13.2X coverage Table S1) were aligned to the Atlantic salmon genome (GenBank: GCA_000233375.4) (Lien *et al.* 2016) using bowtie2 v2.3 (-p2, -sensitive, other parameters at default) (Langmead and Salzberg 2013).

### Association analyses

To reduce Type I error, association analyses were performed using two complementary pathways based on genotype probabilities (*Angsd*, Korneliussen *et al.* 2014) and called genotypes (*Stacks*, Catchen *et al.* 2013).

In *Angsd*, SNP α-value of 1^−6^ (p-value for being variable) was used for a SNP to be processed. In addition, a minimum mapping quality of ten, minimum base score of 20, a minor allele frequency of 0.05 (Roesti *et al.* 2012) and a minimum of presence in 87 individuals were required for a SNP to be called. In addition, SNPs were filtered to exclude loci deviating from Hardy-Weinberg equilibrium with a genome-wide threshold accounting for multiple test correction (-do HWE1, -minHWEpval 0.05 / total number of loci). In order to correct for population stratification, we used *Pcangsd* (Meisner and Albrechtsen 2018) to perform principal component analysis and to obtain a covariance matrix accounting for population structure (one axis was retained). The association study was performed using the -doAsso 2 function (-doMaf 2 -do MajorMinor 1 -do Post 1), corresponding to an association analysis using a quantitative phenotype. Individual LME residuals represented relative migration distances as quantitative trait measures. SNPs that passed all quality filter steps (n = 24330) were ranked based on the likelihood ratio test (LRT) scores. This association test was based on genotype probabilities (see Korneliussen *et al.* 2014) and used a linear regression to give likelihood scores for each SNP individually (see Skotte *et al.* 2012 for more details).

In the alternative pathway, *Stacks* 2.0 (Catchen *et al.* 2013) and its functions *Refmap* and *populations* were used for SNP calling. In addition to the filtering criteria listed above, loci had to present a maximum heterozygosity of 0.5 (Hohenlohe *et al.* 2011), a minor allele frequency of 0.05 (Roesti *et al.* 2012), be present in both populations and in at least eighty-seven individuals to be processed. Finally, the dataset obtained in *Stacks* was tested for Hardy-Weinberg equilibrium in R version 3.4.4 (R Core team 2016) using the *hw.test* function of adegenet (v2.1.1; Jombart 2008). Loci that deviated from Hardy-Weinberg equilibrium (*α* = 0.05/number of loci) were excluded. A compressed mixed linear model (MLM) was fitted using the GAPIT package in R environment (Zhang *et al.* 2010; Lipka *et al.* 2012). 18 264 SNPs were associated with the phenotype scores that were treated as a continuous variable. We corrected for population stratification by using a covariance matrix based on the genetic structure between the populations (“PC.total =1” option in GAPIT). Correction for kinship was performed by obtaining a kinship matrix based on VanRaden method (VanRaden 2008) in GAPIT and using it as a covariance matrix.

The association method in the first pathway (*Angsd*) is based on a linear regression while the second (*Stacks*) relies on a linear mixed model. The linear regression association method takes the uncertainty of the genotypes into account while performing the association and is thus a powerful method than can still control for false positive (Skotte *et al.* 2012). The linear mixed model used called genotypes and is based on the EMMA algorithm (Kang *et al.* 2008) which is arguably the mostly used algorithm used in association studies. As such, the two pathways are based on different assumptions inherent to their methods (Zhang *et al.* 2010; Skotte *et al.* 2012) and overlapping markers, identified in both pathways, are particularly interesting to examine.

### Candidate gene identification

There is a trade-off between identifying true GWAS signals (minimizing false positives) and ignoring more subtle, but real, associations (Type II error) due to low power and multiple testing burden (Kuo 2017). As a consequence, it has been suggested that multiple test corrections are too conservative in a GWAS context (Kuo 2017). For instance, statistically “non-significant” putative candidate genes with sound functional properties have been proposed for different diseases such as essential hypertension (Fowdar *et al.* 2017) or stroke protection in patients with sickle cell anemia (Flanagan *et al.* 2013). Moreover, candidate genes, even when not meeting conservative genome-wide significance after multiple test corrections, can a) indicate potential true and functional association with the studied traits, and b) serve as basis for targeted GWAS (*e.g.*, Rivas *et al.* 2011; Sarzynski *et al.* 2011; Demontis *et al.* 2019).

In order to correct statistical significances for multiple testing, we used a false discovery rate correction (Benjamini and Hochberg 1995) separately for both GWAS approaches. The lack of significant associations after correction for multiple testing can be caused by multiple factors such as markers being too far from the causative variant, limited sample size or variant being at too low frequency in a population to reach genome-wide significance. Therefore, we further identified SNPs that showed the strongest genotype-phenotype associations (top 0.1%) based on both *Angsd* and *GAPIT*, even if these SNPs did not pass significance in multiple testing. Some of these markers can still represent true genotype-phenotype associations particularly in combination with other markers with small effects. As studied markers likely do not represent causative SNPs affecting migration, we identified the closest genes to the observed SNPs based on the Atlantic salmon reference genome.

### Data availability

Supplementary materials are available on Figshare. These include filtered genotypes for both pathways, individual phenotypes, population structure and association results of both analyses. Figure S1 is a picture of the circular channels. Figure S2 is a boxplot of the residuals (*i.e.*, phenotypic scores) between the populations. Figure S3 is a comparison between SNPs obtained for each pipeline. Raw sequence data are deposited on NCBI (PRJNA552287). Movement data and all codes used in this study are available upon request. Supplemental material available at FigShare: https://doi.org/10.25387/g3.7660295.

## Results

### Phenotypic results

According to the LME added with the strain effect, the assumed migratory OUV strain moved significantly farther downstream than the resident VAA strain (*F*_1, 7.34_ = 22.34, *P* < 0.001, average 160.5 km *vs.* 69.1 km), indicating the presence of genetic or epigenetic component affecting migration. The type III fixed effect tests indicated that smolt migration was affected by year (*F*_1, 16.59_ = 7.08, *P* = 0.017) and testing pond (*F*_7, 105.56_ = 2.98, *P* = 0.007) but not by sex of the fish (*F*_1, 179.22_ = 0.40, *P* = 0.526) or the testing pond × year interaction (*F*_7, 105.08_ = 1.26, *P* = 0.278). Because the used fish represented a subset of fish in the smolt migration experiment, these results are technical for the purpose of this work, but representative with respect to population differences due to stratified sampling within each test pond.

### GWAS - Angsd

**Figure 2:**
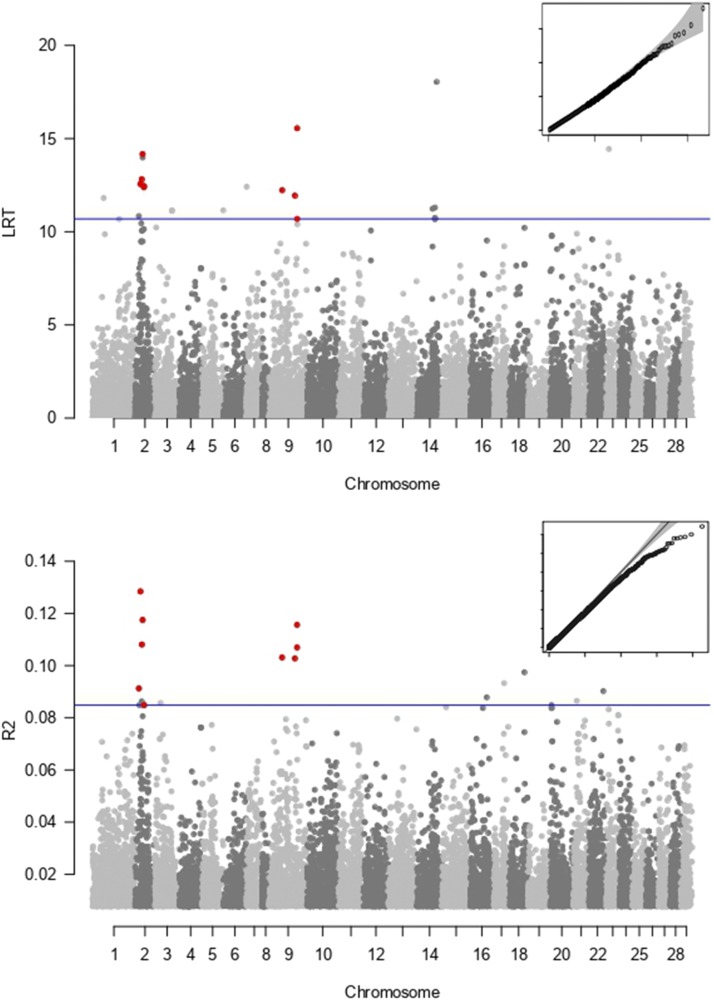
Manhattan plots based on a) 24330 (*Angsd*) and b) 18264 (*Stacks*) SNPs. Overlapping SNPs are marked in red. The horizontal significance line corresponding to the top 0.1% of the SNPs is drawn in blue.

The Q-Q plot showed that there was no inflation in the dataset and the correction for population stratification was adequate (Figure 2). 24430 SNPs were initially obtained in *Angsd*, but 2114 of them did not pass the filtering criteria of the association function (LRT= -999 in *Angsd*).

None of the individual SNPs showed statistically significant association with migration scores after the multiple test correction (FDR) in the final *Angsd* dataset of 22186 SNPs. The top 0.1% percent of markers corresponded to 22 SNPs.

### GWAS – Stacks/GAPIT

Q-Q plots with the *Stacks* dataset showed a conservative pattern with slightly right-skewed distribution indicating that the statistical framework used for correcting for various factors was slightly too conservative (Figure 2). In total, 18 264 SNPs were identified. Similar to *Angsd*, none of the SNPs showed statistically significant individual association with migration distance after the multiple test correction (FDR). The top 0.1% percent of markers corresponded to 18 SNPs.

### Overlap

**Figure 3:**
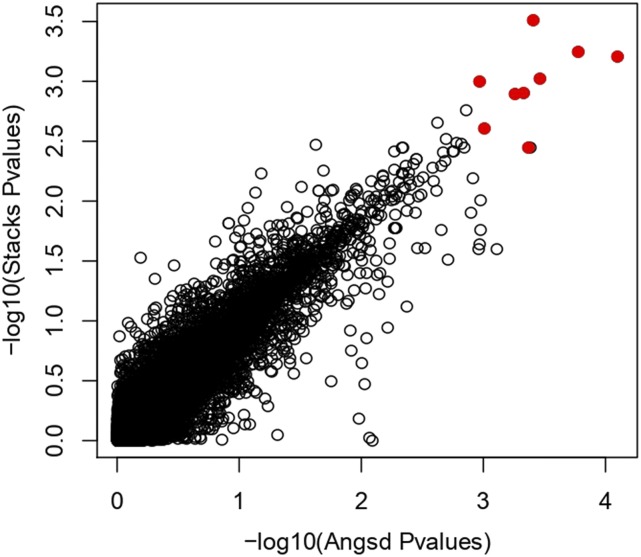
Correlation between P-values of two different GWAS based on different pathways (*Stacks* and *Angsd*). The nine candidate SNPs identified by both association methods are highlighted in red.

87.4% of the 18264 SNPs identified in *Stacks* were found among the 24330 SNPs identified by *Angsd*. The overlap was consistent across chromosomes (Fig. S3). Despite of different analytical approaches, the estimated P-values for *Stacks* and *Angsd* showed very high correlation (Pearson’s r = 0.92, Figure 3). Out of the top 0.1% markers of both pipelines (24 and 18 SNPs respectively), nine SNPs were overlapping (Table 1). These SNPs mapped to two chromosomes (Chr 2 and 9) based on the Atlantic salmon reference genome (Figure 2, Table 1). Four SNPs were found within genes, while five SNPs were found from 2565 to 48527 bp from the closest known coding regions.

**Table 1:** Candidate migration genes and protein products identified by both *Stacks* and *Angsd*. Chromosome number refers to Atlantic salmon genome

Chromosome	SNP position	Distance to closest gene (bp)	Predicted protein	Gene symbol
2	13725458	48527 - 3′end	FXYD domain containing ion transport regulator 5a precursor	FXYD5A
2	19563669	2565 – 3′end	flotillin 1	FLOT1
2	24989435	0	progestin and adipoQ receptor family member 6-like isoform X2	PAQR6
2	27622691	1460 – 3′end	free fatty acid receptor 2-like	FFAR2
2	32550984	14430 – 5′end	plectin-like isoform X14	PLEC
9	48392079	16589 – 5′end	protocadherin gamma-A11-like	PCDHGA11
9	96081278	0	ATP-binding cassette sub-family F member 3-like	ABCF3
9	103885594	0	limbic system-associated membrane protein-like isoform X	LSAMP
9	103988881	0	limbic system-associated membrane protein-like isoform X	LSAMP

## Discussion

This common garden study demonstrated divergent migratory patterns between two populations but did not find genome-wide significant SNPs with strong effect on migratory distance. However, smolt migration distance was individually highly repeatable between the test years and showed strong differences between two populations, which suggests that the trait could be heritable, as repeatable behavioral traits typically show reasonably high heritability (Dochtermann *et al.* 2015). The two statistical analysis pipelines used for association analysis revealed nine shared SNPs that could potentially affect migratory behavior but none of them overlapped with outlier SNPs identified by Lemopoulos *et al.* (2018). Together these results suggest that the migration distance in brown trout has underlying genetic components implying evolvability, but the genetic architecture is likely multigenic and the potentially existing regulatory genes with major effects remain to be identified.

Nine migration-associated candidate SNPs were identified by both bioinformatic methods. These markers mapped to two separate chromosomes in Atlantic salmon genome (Chr 2 and 9) and clustered together in relatively narrow genomic regions (Figure 2; Chr 2: 18.8 Mb, Chr 9: 55.6 Mb) indicating that the observed signals most likely reflect real associations, rather than random noise. According to a previous linkage map (Leitwein *et al.* 2017b), these chromosomes corresponded to brown trout linkage groups 20, 34 and 23. Within the 18.8 Mb region in chromosome 2, 559 genes are annotated in the Atlantic salmon genome. Three of the markers identified in chromosome 9 were mapped within a 7.6 Mb region consisting of 196 genes.

The nine migration-associated candidate SNPs mapped adjacent to eight genes involved in diverse biological functions relevant to migration (Table 1). For example, FYXDa gene, which located 48527 bp from the migration-associated SNP, is an important ion transporter that regulates Na^+^/K^+^ ATP-ase pumps. The function of these pumps is well documented in salmonids (*e.g.*, Zaugg 1982; Nielsen *et al.* 1999; Larsen *et al.* 2008) as they play significant role for osmoregulation. Similarly, PLEC plays a role in coping with osmotic stress (Osmanagic-Myers *et al.* 2006) and has been shown to be upregulated in seawater-exposed whitefish *Coregonus lavaretus* (Papakostas *et al.* 2012) while ABCF3 is differentially expressed in gills of freshwater or seawater exposed rainbow trout, demonstrating its putative role in acclimation to seawater (Leguen *et al.* 2015). Among the other candidate genes, LSAMP is part of the limbic system, which itself is important for memory and spatial orientation, via the hypothalamus (Portavella and Vargas 2005; Catani *et al.* 2013). Nervous system development and memory are key factors in migratory behavior and especially for homing, as shown by differential gene expression and DNA methylation patterns in *O. mykiss* (McKinney *et al.* 2015; Baerwald *et al.* 2016). Moreover, PAQR6 is also expressed in the hypothalamus and other regions of the brain (Thomas and Pang 2012; Morini *et al.* 2017). The hypothalamus and thalamus regions could play an important role in the migratory behavior of salmonids through different pathways. In particular, circadian rhythms (see Prince *et al.* 2017; Pritchard *et al.* 2018), osmoregulatory mechanisms (Prunet *et al.* 1989; Hourdry 1995) and memory (Carruth *et al.* 2002; Ebbesson *et al.* 2003), are all potentially (inter)linked (Kim *et al.* 2015) within the hypothalamic region. Finally, PCDHGA11 is part of the cadherin gene family that has been identified both in a previous study comparing resident and migratory brown trout (Lemopoulos *et al.* 2018), and in rainbow trout (Hale *et al.* 2013; Baerwald *et al.* 2016). Cadherins play an important role in brain function and our results, together with earlier findings, support the role of cadherins affecting salmonid migratory behavior (Lemopoulos *et al.* 2018). The functions of the last two candidate genes (FFAR2, FLOT1) are linked to several diverse cellular processes but also exhibit plausible links to migratory behavior. In particular, FFAR2 is involved in lipid metabolism and autoimmune functions (Bjursell *et al.* 2011; D’Souza *et al.* 2017), while FLOT1 is involved in signal transduction and vesicular transport processes in the brain (Bickel *et al.* 1997; Volonté *et al.* 1999) corroborating earlier studies suggesting that lipid metabolism (Boel *et al.* 2014), immunity (Sutherland *et al.* 2014) and brain-related functions (McKinney *et al.* 2015) may play key roles in affecting migratory strategies.

The present study aligns with the previous ones by suggesting that the migration propensity is likely a polygenic trait in brown trout (Lemopoulos *et al.* 2018; Ferguson *et al.* 2019). This is plausible given that smolt migration is associated with changes in the expression of multitude of genes (*e.g.*, Giger *et al.* 2006; Seear *et al.* 2010; Hecht *et al.* 2014) with multiple physiological functions, such as osmoregulation (Hecht *et al.* 2014), immunity (Sutherland *et al.* 2014), and growth (Boel *et al.* 2014). Surprisingly, while fish ascending toward spawning grounds often have female-biased sex-ratio (Dodson *et al.* 2013), individual sex was not found to be significant with regard to migration distance in our experiment. This could potentially reflect population specific patterns or suggest that rather demographic effects than true biological differences between the sexes drive the patterns observed in nature. As we included individuals originating from both resident and migratory populations, population structure was inherently confounded with the individual traits inducing migration. While the analysis was performed at the individual level, the removal of population stratification from the GWAS could have led to discarding functionally relevant population-specific variation. Thus, further experiments using multiple populations or populations with mixed origin should be performed to increase the likelihood of finding a signal inherent to species-specific rather than population-specific genes.

Similar to many other studies on non-model species, our experimental design likely suffered from non-optimal statistical power due to limited sample size and limited number of markers. On the other hand, it plausible that the multiple testing correction was too conservative to single out loci with real but small biological effects (*e.g.*, Lantieri *et al.* 2010; Fowdar *et al.* 2017). Genomic data typically consist of thousands of markers tested for potential association and in most cases, very few - if any - markers reach the required level of statistical likelihood for a significant effect (*e.g.*, Correa *et al.* 2017; Barría *et al.* 2018). This is particularly true when a) the studied traits are under the control of multiple loci of small effects (*i.e.*, a polygenic trait, see Boyle *et al.* 2017) and b) when the studied populations are structured in terms of the trait of interest (Zhao *et al.* 2011), as in our case. As an alternative strategy, we searched for overlapping candidate genes that were identified as potentially interesting by two analytical approaches (*Stacks* and *Angsd*). Depending on data structure and method assumptions different approaches can perform differently (Wojcik *et al.* 2015; Zhu *et al.* 2018), and thus combining methods can potentially reduce both Type I and Type II error, analogously to genome-scans (Vasemägi and Primmer 2005). In this regard, the association results from *Stacks* and *Angsd* were similar, but not identical, demonstrating the usefulness of complementary statistical approaches in GWAS (Figure 3).

Compared to previous comparative work (Lemopoulos *et al.* 2018), none of the top migration-associated SNPs were overlapping between studies. Given that different enzymes were used in the RAD protocol (*Sbf1 vs. PstI-BamHI* respectively) this is hardly surprising, as these studies analyzed to large extent non-overlapping parts of brown trout genome. Yet, the lack of statistically significant individual SNPs after FDR correction is not valid counterevidence for migration being genetically influenced, because SNPs identified using RADSeq cover only a small proportion of the whole genome. Thus, it is possible that many causative variants affecting migration were not captured, given the low level of linkage disequilibrium in brown trout (Ahmad *et al.* 2018). To address this issue, the use of hundreds of thousands rather than tens of thousands of markers screened using genome-wide SNP arrays, high frequency RADseq (*e.g.*, using four cutters instead of six or eight cutter enzymes) or whole-genome sequencing would be necessary.

Our study shows how brown trout individuals and populations differing in their migration strategies could bear genetic signatures associated with their life-history. This alone is a valuable result for management and conservation purposes, as it indicates that ecotypes should be managed differently in order to maintain the life-history diversity (Waples and Lindley 2018). These results are also valuable in gene-targeted conservation plans perspective. While clear-cut diagnostic candidate genes are still needed for sound conservation plans and application for conservation practitioners (Shafer *et al.* 2015; Kardos and Shafer 2018), these results expand the existing knowledge of brown trout migration and can thus serve as a basis for future conservation-oriented studies.

To conclude, our study demonstrates that migration in brown trout has a genetic or epigenetic component but does not fully resolve the mechanistic and causal pathways for variation in migration tendency. By linking telemetry in common garden with genomic data, we identified two promising genomic regions and eight candidate genes potentially associated with migratory behavior. However, additional testing using higher number of SNPs and analysis of inter-population hybrids is still needed for validation of the putative association signals and for better understanding of the molecular function and adaptive significance of the identified candidate genes.
